# Mindfulness, empathy and moral sensitivity in nurses: a structural equation modeling analysis

**DOI:** 10.1186/s12912-022-00912-3

**Published:** 2022-05-27

**Authors:** Yasser Rezapour-Mirsaleh, Mahdi Aghabagheri, Azadeh Choobforoushzadeh, Azra Mohammadpanah Ardakan

**Affiliations:** 1 Department of Counseling, Faculty of Humanities and Social Sciences, Ardakan University, P.O. Box184, Ayatollah Khatami Blv., Ardakan, Yazd, Iran; 2 Nursing Meybod School, Shahid Sadoughi University of Medical Sciences, Yazd, Iran; 3 Department of Psychology, Faculty of Humanities and Social Sciences, Ardakan University, Ardakan, Iran

**Keywords:** Mindfulness, Empathy, Moral sensitivity, Nurses

## Abstract

**Background:**

Ethical issues may pose challenges to nurses; moral sensitivity can help them to overcome these challenges. Identifying variables related to moral sensitivity can help in planning to increase nurses’ moral sensitivity. This study aimed to investigate the relationship among mindfulness, empathy, and moral sensitivity in a sample of nurses.

**Methods:**

In the present study, a cross-sectional design utilizing Structural Equation Modeling (*SEM*) was conducted. The nurses in a private hospital in Yazd, Iran, were invited to participate in the study (*n*=162) using simple random sampling. In order to gather the data, the Freiburg’s mindfulness inventory, moral sensitivity questionnaire, and revised Jefferson’s empathy scale were used. The hypothesized model was analyzed by SEM.

**Results:**

The results show that Mindfulness (*β*=0.41, *t*=5.53, *p*<0.01) and empathy (*β*=0.52, *t*=6.77, *p*<0.01) had a significant direct effect on moral sensitivity. However, mindfulness had an indirect effect on nurses’ moral sensitivity via empathy improvement (*z*= 6.25, *p*<.01).

**Conclusion:**

Empathy played a significant mediating role in the relationship between mindfulness and moral sensitivity, so mindfulness-based interventions with an emphasis on empathy may provide an opportunity to increase moral sensitivity in nurses.

## Background

Nurses often encounter challenging moral issues that put them in situations where they have to make difficult decisions. They are required to be sensitive on moral issues related to their responsibilities, decision-making processes about patients, management problems, and challenges in clinical environments. Nurses who can not solve moral problems in the workplace suffer from moral distress that can affect the quality of their work and affect patient care [1].

Moral sensitivity as a concept includes deeds, wills, feelings, and comprehensions compassing of various categories which is not easy to comprehensively defined [2]. Nurses are required to have ethical sensitivity and skills to recognize their own and others’ values and beliefs in order to make moral decisions in caring situations; therefore, moral sensitivity is defined as an ability to recognize patients’ vulnerabilities and predict the consequences of moral decisions in patients, especially in cases where there is an moral ambiguity. Moral sensitivity is such a value that can be so fruitful in a challenging situation and that can lead to nurses awareness raising. In fact, moral sensitivity is an ability to recognize a moral problem and notice moral decision consequences [3]. It is a characteristic helping a nurse to recognize the moral conflicts of his career, appropriate inferring on patient problem, and making aware of the decision consequences on patients. In sum, mentioned aspects can lead to a more proper moral care. In the same way, moral sensitivity improves a skill for a nurse to solve a value conflict more appropriately; regarding, empathy is a mandatory prerequisite for it [4]; so that, a more skillful empathetic nurse, a more successful one in moral sensitivity.

### Moral sensitivity and empathy

Concept of empathy in a therapeutic conceptualization is defined as a proper interpretation based on internal frame of reference of others, so in an empathic relationship a therapist should inference emotions exactly like the client experiences [5]. Empathy is an ability to identify a person’s feelings and put ourselves in that person situation; in other words, empathy is considering different issues from other person viewpoint [6]. Morality and empathy are intertwined with human essence; in this regard, experimental findings in behavioral and social sciences show that there is a relationship between empathy and moral sensitivity; however, in all cases, there is not a direct effect from empathy to moral sensitivity. Sometimes, an empathetic person cannot recognize and evaluate the client situation without considering the situation involved and moral issues [7]. Empathy is one of the most important factors in considering moral issues and can improves the moral sensitivity [8, 9]. There was a positive relationship among empathy, nurses career morality consideration, and moral sensitivity [10]. Without empathy, the appropriate recognition of client needs and problems is impossible for nurses, and then they cannot do their moral responsibility appropriately. There are positive correlation between empathy, interpersonal competence, and ideal nurse attributes [11]; and empathy causes the reduction of career faults and defies career responsibilities in nurses and also reduces the client complaints and dissatisfaction of care services [12].

### Moral sensitivity and mindfulness

In addition to empathy, one of the other variables which may improve the moral sensitivity of nurses is mindfulness ability. Mindfulness ability is considering the present situation without any prejudice [13]. Prejudiced opinion that does not take to account the real reason or experience is exactly the opposite concept of mindfulness and it can be called as non-attachment. One of the main aspects of mindfulness is meditative focus which means concentrating on the present events and ignoring the past and future incidents [14]. Mindfulness influence moral decision-making in two ways; first, it can lead to awareness-raising of the situation and this awareness-raising can result to a more rational decision-making, ignoring the negative beliefs and better managing the negative feelings [15]. This unbiased decision-making can lead us for a more moral behavior [16]. Second, mindfulness improves self-awareness and then self-awareness impedes the immoral behavior. Experimental findings show that a more self-aware person is more honest [15, 16].

### Hypothesized model

**Fig. 1:**
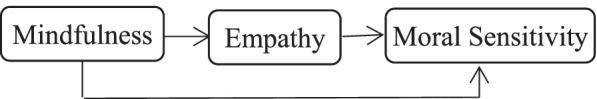
Hypothesized model

Not only does mindfulness influence on moral sensitivity directly, but probably it can influence on empathy indirectly and then raise a nurse moral sensitivity. Mindfulness can lead to concentration and mindfulness mediation is a way to make empathy in Buddhism School; regarding, some probes reveal that mindfulness can result to empathy improvement [17, 18]. Mindfulness can lead to the experiment enrichment by empathically acknowledging, expand the vision horizons and critical examination of personal biases which consequently results in improving empathy [19]. In a study, focusing on health care providers, findings show that mindfulness intervention can improve empathy; If mindfulness and attention to here and now are taught to nurses, they can be more empathetic with patients and then provide a better care [20]. Considering the aforesaid illuminations, the relationship between mindfulness, empathy, and moral sensitivity can be illustrated as follows in figure 1.

### Purpose

Considering the significance of moral sensitivity in nurses care quality, the investigation of influential variables is paramount of importance. The present study aimed to investigate the in relationship among mindfulness, empathy and moral sensitivity in nurses. According to the purpose, three hypotheses were raised:

1. Mindfulness has a direct effect on the moral sensitivity of nurses.
2. Empathy has a direct effect on the moral sensitivity of nurses.
3. Mindfulness through empathy has an indirect effect on the moral sensitivity of nurses.

## Methods

### Sample and procedure

A cross-sectional correlational study design, involving 162 nurses in a hospital in Yazd City, Iran, was conducted. The study was adhered to the STROBE guideline for cross-sectional studies. Only one hospital was selected because the hospital climate could have an impact on the nurses’ moral sensitivity. In total, there were 312 employed nurses in this hospital; the sample size was estimated to be 172 nurses based on Cochran’s sampling formula. The participants were selected by simple random sampling method; first, a list of all nurses working in the hospital was prepared and each nurse was allocated by a number, then the participants were identified using a table of random numbers. A random number table is a series of digits (0 to 9) arranged randomly in rows and columns and is used to randomly select numbers. Selected nurses who did not agree to participate in the study, did not meet the inclusion criteria or had the exclusion criteria, were replaced by another participant using the same way by table of random numbers. However, finally, after selecting the nurses and distributing the questionnaires, the data of 10 participants were distorted and could not be analyzed.

### Data collection

Data collection was carried out from 8 to 27 June 2019. From 172 distributed questionnaires, 162 (94.2%) were valid. Inclusion criteria were as follows: at least 2 years of work experience, shift rotation, age (25-50), nurses with a Bachelor’s degree and the only exclusion criteria was mental disorder. How to answer the questionnaires was explained to the participants. The scales were completed individually and the participants’ questions were answered so that there would be no ambiguity.

### Measures

#### Moral sensitivity scale

Lützén et al developed a short form questionnaire named as "moral sensitivity test" in order to evaluate moral sensitivity during providing medical care [21]. The scale consisted of 9 items that were divided into three subscales of sense of moral burden, moral strength and moral responsibility; scoring was done in 6-range Likert Scale (1= strongly disagree, 6=strongly agree). Moral burden (include 4 items) includes problems and situations that arise from conflicts with moral values; however, on this scale, this is not a negative concept and indicates the nurse’s readiness to face the moral burden. Moral strength (include 3 items) means having the courage and ability to provide reasoning to justify patients for actions that are moral. Moral responsibility (include 2 items) means a commitment to do tasks according to moral rules and values [21]. Scores ranged from 9 to 54 and higher scores indicate more moral sensitivity. Validity and reliability of that was approved. the subscales named are sense of moral burden, moral strength, and moral responsibility, which were determined by factor analysis [21]. Validity and reliability of the Persian version of the long-form of this questionnaire have been approved by a sample of Iranian nurses [22]. The Cronbach’s alpha of the scale in the present study was .94.

#### Jeferson scale of empathy

The Jeferson Scale of Empathy is a 20-item scale was scored in 7-range Likert Scale from 1 (strongly disagree) to 7 (strongly agree) [23]. This scale was include three subscales named as perspective taking, compassionate care, standing in a patient shoes. Perspective taking means the nurse’s view from the patient’s perspective and nurses’ understanding of a patient’s perspective. Compassionate care refers to paying attention to patients’ emotions in caring so that the nurse has empathetic concern for the patient while maintaining a reasonable distance to keep emotional balance. Standing in a patient shoes means thinking like a patient and understanding the patient’s experiences and emotions exactly as he or she experiences them [23]. Scores range from 20 to 140 and higher scores indicate more empathy. Validity and reliability of the scale was approved [23]. Validity and reliability of the Persian version of the scale have been approved by a sample of Iranian nurses [24]. The Cronbach’s alpha of the scale in the present study was .94.

#### Freiburg mindfulness inventory

Freiburg mindfulness inventory includes 14-items which categorized in two subscales as presence and acceptance [25]. This scale was scored in 4-point Likert Scale (1 =rarely, 4= always). Presence means present inner experience and acceptance of pleasant or unpleasant inner experience in the here and now. Acceptance refers to the desire and readiness to be exposed to pleasant and unpleasant experiences; in other words, acceptance means non-judgmental acceptance of experiences [25]. Scores range from 14 to 56 and higher scores indicate more mindfulness. The validity and reliability of the Persian version was approved [26]. The Cronbach’s alpha of the scale in the present study was .84.

### Data analysis

The gathered data utilizing Pearson correlation, and Structural Equation Modeling (SEM) were analyzed by SPSS Version 20 and SmartPLS Version 2. Missing data were replaced with that participant’s mean on the subscale to which the missing item belonged. Path analysis was used by partial least squares structural equation modeling, SmartPLS [27], which is a good method for evaluating simultaneously relationships among multiple latent variables and mediating effect [28]. PLS is based on regression analysis that maximized The *R*^*2*^ values of the dependent variable(s). Because the sample size in this study was small, PLS was a better approach to investigate the mediating role than other techniques [28]. For investigate hypothesis model, we used 3 criteria: First, reliability and validity of the measures were evaluated. Reliability analyzed by Cronbach alpha and composite reliability (CR) which should be higher than 0.70; and indicator’s loadings which should be higher than 0.5. Convergent validity evaluated using the average variance extracted (AVE), which should be higher than 0.50. Discriminant validity evaluated by comparing square root AVE of the latent construct to the correlation between that construct and other latent construct, and by indicators cross-loadings [28]. Second, the significant of path coefficients assessed using bootstrapping approach (t-values should be higher than 1.96 for two-tailed test) and effect sizes evaluated by *R*^*2*^ values, [28]. Finally, global fit analysis for PLS modeling was evaluated using goodness-of-fit (GoF =$\sqrt{\overline{AVE }*{R}^{2}}$) which 0.1, 0.25 and 0.36 are small, medium and large values for global fitness of the model respectively [29]. According to ‘10-times rule’ method [30] the minimum sample size for PLS-SEM should be greater than 10 times the maximum number of inner or outer model links pointing at any latent variable in the model. Because there were 11 links to latent variables in the proposed model, at least 110 samples were required for structural equations analysis. However, according to the population size and Cochran’s formula, 172 nurses were sampled and finally the data of 162 participants could be analyzed. Therefore, according to 10-times rule, the sample size is suitable for analyzing using the partial least squares (PLS) method. Klomogorov-Smirnov test was used to evaluate the normality of the distribution of scores. The results showed that the test statistics are significant for all three variables of moral sensitivity, empathy and mindfulness and the distribution of scores of these variables is not normal. A significance level of 0.05 was used for all tests.

## Results

### Participants’ characteristics

The participants’ characteristics are showed in table 1. Most of the participants were women (63.6%), and the most were married nurses (78.4%). The participants majority were nurses employed in Internal-Surgical (35.8%) and Emergency (21.6%) wards. Participants age and work experience were reported as follows, respectively: M=33.70, SD=6.47; M=8.21, SD=5.54.

**Table 1:** Frequencies for demographic characteristics

Frequencies		n	%
Sex	Female	103	63.6
	Male	59	36.4
Marital Status	Married	127	78.4
	Single	35	21.6
Clinical Unit Type	Internal-Surgical	58	35.8
	Emergency	35	21.6
	Delivery	23	14.2
	NICU	11	6.8
	CCU & ICU	24	14.8
	Radiology & Dialyze	11	6.8

### Descriptive Statistics

Mean, standard deviation, and correlation between the study variables were showed in table 2. Skewness and kurtosis range of total scores were between -0.50 to 0.43 and -0.89 to -0.12 respectively. The P-P plots showed distribution of the data was not normal but can be considered approximately normal. The subscales of any variable are strongly correlated with each other (Table 2). The total scores of empathy, moral sensitivity, and mindfulness were also strongly and positively associated (*p*<0.01) (Table 2).

**Table 2:** Mean, standard deviation and correlation between study variables

Study variables		M	SD	1	2	3	4	5	6	7	8	9	10
Empathy	1. perspective taking	44.41	16.98	1									
	2.compassionate care	25.07	11.38	.47^*^	1								
	3. standing in the patient’s shoes	6.26	3.35	.41^*^	.79^*^	1							
	4. Total score	75.73	26.77	.88^*^	.82^*^	.72^*^	1						
Moral Sensitivity	5. sense of moral burden	17.19	6.60	.75^*^	.50^*^	.42^*^	.74^*^	1					
	6. moral strength	14.41	4.89	.83^*^	.37^*^	.28^*^	.72^*^	.79^*^	1				
	7. moral responsibility	9.52	3.46	.83^*^	.40^*^	.32^*^	.74^*^	.79^*^	.90^*^	1			
	8. Total score	41.12	14.05	.84^*^	.46^*^	.37^*^	.78^*^	.94^*^	.94^*^	.93^*^	1		
Mindfulness	9. presence	12.67	3.49	.57^*^	.43^*^	.42^*^	.61^*^	.72^*^	.64^*^	.64^*^	.72^*^	1	
	10. acceptance	21.42	5.19	.52^*^	.34^*^	.27^*^	.51^*^	.60^*^	.54^*^	.53^*^	.60^*^	.59^*^	1
	11. Total score	34.09	7.78	.61^*^	.42^*^	.37^*^	.61^*^	.72^*^	.65^*^	.64^*^	.72^*^	.84^*^	.93^*^

^*^p<.001

### Test of the hypothesized model

#### Reliability and validity of the measures

All factor loadings of the empathy and moral sensitivity indicators were above 0.7 which satisfies fit criterions [28]. One indicator of the acceptance (from mindfulness latent construct) was less than 0.5 and was deleted (item no. 13). The other indicators of the mindfulness were above 0.5 (7 indicators were above 0.7) that meet minimum cutoff value; Factor loading which are above 0.5 and statistically significant can be maintained in the model [31]. The Cronbach’s alphas and CR of all latent variables were higher than 0.70 (Table 3) which were consistent with Hair et al fit recommendation [31]. Therefore, the results showed reliability and internal consistency of the indicators were appropriate (Table 3).

**Table 3:** Reliability, validity & R^2^ of the model

Study variables		Number of Items	α	CR	AVE	R^2^
Empathy	1. perspective taking	10	.95	.96	.70	.80
	2. compassionate care	7	.90	.92	.58	.66
	3. standing in the patient’s shoes	3	.76	.89	.81	.51
	4. Total	20	.94	.85	.66	.44
Moral Sensitivity	5. sense of moral burden	4	.88	.92	.74	.88
	6. moral strength	3	.85	.91	.78	.90
	7. moral responsibility	2	.83	.92	.85	.88
	8. Total	9	.94	.96	.89	.71
Mindfulness	9. presence	5	.77	.78	.53	.78
	10. acceptance	6 (3 items were deleted)	.79	.81	.50	.81
	11. Total	14	.85	.88	.79	--

To evaluate the convergent validity of the latent construct, the average variance extracted (AVE) were calculated; all of the AVE except acceptance were higher than 0.5. Two indicators from acceptance which had lower AVE than other indicators (items no. 11 and 14) were deleted and then AVE of this latent construct reached to 0.5. In general, results revealed convergent validity of the measurement model was confirmed. The discriminant validity was analyzed using two criterions: a) factor loadings of each indicator were higher than its cross-loadings [28], and b) square root of each latent variable’ AVE was higher than the correlation between that construct and other latent construct according to Fornell-Larcker criterion [32]. All of the square roots of the AVEs were higher than the correlation between that construct and other latent construct except compassionate care (from the empathy) and moral strength (from moral sensitivity). However, AVE in these latent constructs was higher than with only one other latent construct and it cannot be said that discriminant validity has been completely violated (table 4).

**Table 4:** Intercorrelations of Latent Variables

Study variables		1	2	3	4	5	6	7	8
Empathy	1. perspective taking	**.84**							
	2.compassionate care	.48	**.76**						
	3. standing in the patient’s shoes	.41	.79	**.90**					
Moral Sensitivity	4. sense of moral burden	.75	.50	.42	**.86**				
	5. moral strength	.83	.38	.28	.80	**.88**			
	6. moral responsibility	.83	.40	.31	.80	.91	**.92**		
Mindfulness	7. presence	.58	.44	.42	.72	.64	.65	**.73**	
	8. acceptance	.55	.41	.36	.62	.57	.56	.59	**.71**

#### Estimating effect of mindfulness and empathy on moral sensitivity

**Fig. 2:**
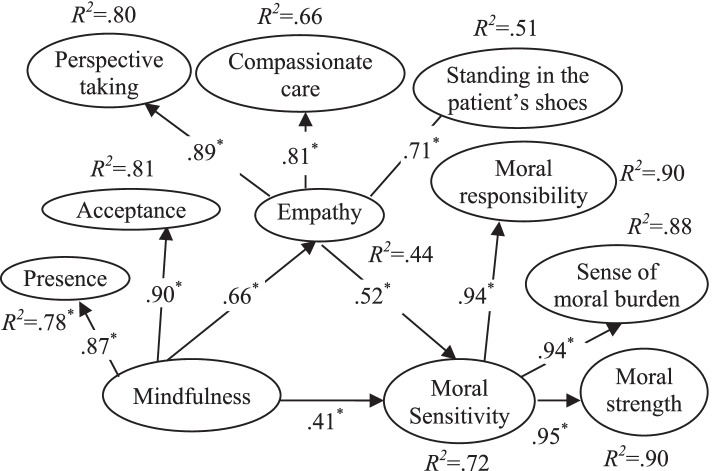
Final model

Conceptual model was analyzed using PLS-SEM according bootstrapping approach in SmartPLS, Version 2. The results revealed that mindfulness had a main effect on empathy (*β*=0.66, *t*=11.07, *p*<0.01) and moral sensitivity (*β*=0.41, *t*=5.53, *p*<0.01) which accounted for 43.6% and 16.8% of the variance in the direct path respectively. Also, empathy had a direct effect on moral sensitivity (*β*=0.52, *t*=6.77, *p*<0.01) which accounted for 27% of the variance in the hypothesized direction (Fig 2). Indeed, nurses who have higher score in mindfulness reported higher empathy and moral sensitivity and higher score in empathy accompanied with higher score in moral sensitivity. All *R*^*2*^ value of the endogenous constructs (except empathy, *R*^*2*^=0.44), were higher than 0.5. 0.75, 0.50 and 0.25 values can be considered substantial, moderate and weak respectively (Fig 2). Finally, bootstrapping analysis showed all t values in both of measurement model and structural model are significant (*p*<0.01).

Global criterion goodness-of-fit estimate for structural modeling which was GoF= 0.72. 0.01, 0.25 and 0.36 values are small, medium and large respectively for GoF according to Wetzels et al recommendation [29].

#### Indirect effect

For evaluating the mediating role of empathy in the relationship between mindfulness and moral sensitivity in nurses, we analyzed the model once including empathy and once excluding it. The main effect of mindfulness on moral sensitivity when empathy excluded from model was significant (*β*=0.75, *t*=20.22, *p*<0.01). On the other hand, the direct path from mindfulness on moral sensitivity when empathy include in the model decreased but remained significant (*β*=0.41, *t*=5.53, *p*<0.01; Fig 2). Therefore, the results revealed empathy plays a mediating role in the relationship between mindfulness and moral sensitivity in the nurses. On the other hand, not only mindfulness has a direct effect on moral sensitivity, but it can have an indirect effect on moral sensitivity via empathy improvement. Sobel test conducted used to calculate the indirect path coefficient. Standard error for mindfulness to empathy path was .053 and for empathy to moral sensitivity path was .072. Considering the path coefficients and standard errors, the value of Sobel was z= 6.25. Values higher than 2.58 are significant at the level of *p* <.01

## Discussion

The findings revealed empathy and mindfulness have direct effect on moral sensitivity. In addition, mindfulness via empathy can increase the moral sensitivity indirectly. This result is in line with the findings of previous studies [7, 16, 33]. Empathy is sometimes epistemologically necessary for recognizing the right action. It also motivates one to do the right thing and identifies the moral worth of doing the right thing [34]. Nonetheless, empathy is not always a direct way for a moral behavior and it can indirectly influence on morality. There are a positive relationship between empathy and nurses’ career morality consideration, and moral sensitivity. The appropriate conduction of moral issues and sensitivities in nurses is related to their empathy as a psychological characteristics [10]. We always need empathy to make the right decisions, especially in relation to others. We need to understand the situation of others and put ourselves in their shoes so that the decision we make does not hurt them. In many cases, what makes these decisions so difficult is that it is not clear how the people involved will be affected by our choices. But empathy with others can help us become more aware and recognize which action is morally right [34]. Empathy plays a vital role in personal commitments and social interactions [7]. In the other hand, moral behavior representation and sensitivity to moral affairs in care situations are in need of identifying client feelings and pains. Considering the nurses vivid role, empathy is an ability improving clients verbal and nonverbal communication; therefore, empathy characteristics can improve moral behaviors and sensitivity to moral affairs among nurses; because empathy is the social facet representation of personality in which it can pave the way for objectivity of moral values in interrelationships like nurse and client [33]. In addition to medical settings, there is evidence in other settings that empathy can affect moral sensitivity [35]. The path of empathy to moral sensitivity is not always one-sided, and sometimes moral sensitivity can also affect empathy in nurses [36].

The findings of this study showed that mindfulness, like empathy, have a positive effect on nurses’ moral sensitivity. In the case of relationship between mindfulness and moral sensitivity, it can be expressed that mindfulness is a concept related to some awareness confined to here and now, and not only is it included unbiased judgment, but it needs consciousness, concentration, and open mind in nurses [19]. The evidence shows that individuals who are more mindful, have better moral decisions, are more committed to ethics, and have more ethical behaviors [16].

In this regard, mindfulness is defined as raised concentration and awareness of what happens here and now. Mindfulness and morality are intertwined. Mindfulness is a way to communicate with life more effectively and it can make life meaningful, and through this way, it can improve moral sensitivity [16]. Consequently, it causes some changes in a person and it can change him to a more sensitive one toward moralities. Mindful people usually make better moral decisions because they are aware of what is going on around them and can analyze issues better; Identifying the right actions and facing moral challenges is also more common in mindful people because they have a better focus on moral values [37]. Evidences show that in medical settings, moral issues can be practiced mindfully. A mindful concentration on ethical challenges leads to better performance of health care providers [38, 39].

Findings showed there is a positive significant relationship between mindfulness and moral sensitivity; it means by the increase of mindfulness, moral sensitivity would increase as well. Considering the findings, mindfulness is one the predicators of moral sensitivity. Mindful persons usually possess such an ability to comprehend their feelings deeply and their acceptance as well as the comprehension for the subtle issues. In general, they are self-aware, positive, and reliant about their own and they are successful in considering the personal, social, and occupational issues [40]. Mindfulness signifies a complicated method to direct consideration and facilitates present cognizance and it can be generally conceptualized as refining consideration and emotional processes [25]. Mindfulness can increase moral motivations and behaviors through various ways such as increasing awareness of oneself and others, reducing behavioral and emotional difficulties, strengthening moral support agents such as altruism, love, compassion, consciousness, and empathy. Because moral reasoning is accompanied by awareness, mindfulness can improve moral reasoning by raising awareness at the moment. Evidences revealed that there is a negative relationship between mindfulness practice and health providers burnouts and compassionate fatigue and there is a positive relationship between mindfulness practice and well-being [41].

In line with the mediator role of empathy in the relationship between mindfulness and moral sensitivity, it can be declared that mindfulness influences on empathy via awareness raising. Evidence showed empathy played a mediating role in the relationship of mindfulness and therapeutic alliance [42]. Practicing mindfulness in health care provider generally not only leads to reduces the probability of responding to clients in a way that jeopardizes therapeutic relationships but also improve their ability to have a more effective relationship with clients through increasing awareness, accountability and accountability [43]. The evidence show that nursing students who were more skilled in mindfulness had better empathy with their clients via helping them express their feelings and excitement more easily and paying more attention to their distress [19]. Because mindfulness raise awareness and acceptance of self and others, then leads to a better empathy with others [17]. Nurses with a higher mindfulness are not affected by the negative emotions of clients in the empathy process. therefore, clients distress does not transfer to them and they can have a more effective therapeutic relationship with clients; regarding, they are prone to make a more sensitive decisions [42]. Mindfulness leads to a shift in the ego-centric frame of reference to other-centric frame of reference. Therefore, it creates the conditions for the person to consider the welfare of others alongside welfare of her/himself, as a result, to have more empathy with others and to make better moral decisions in the face of others [43]. In line with the results, a study showed that empathy has a mediating role in the relationship between mindfulness and engagement in nurses. One of the characteristics of proper engagement in nurses is paying attention to moral issues. Therefore, it can be conclude that mindfulness through empathy can increase attention to ethical issues in the medical environment [44].

## Conclusion

In accordance with the findings of the study, it can be concluded that, not only mindfulness and empathy can affect directly on moral sensitivity of a nurse, but mindfulness can improve the nurse empathy and regarding indirectly improve moral sensitivity of the nurses. In other words, nurses who were more moral sensitive, reported more empathy, and more mindfulness, were able to communicate with clients "instantly here and now", have more empathy with patients and, as a result, make better moral decisions in care situations. As a result, empathy in the relationship between mindfulness and moral sensitivity plays the role of a mediator. Therefore, via mindfulness improvement in nurses, it is possible to raise their moral sensitivity; considering the empathy role as a mediator, it is recommended mindfulness improvement is coincided with empathy. Finally, because the indirect effect of mindfulness on the moral sensitivity of the nurses through empathy was greater than its direct impact, it can be concluded that, regardless of empathy, nursing mindfulness exercises cannot be beneficial to improving moral sensitivity. As mentioned, empathy training can be preferably done if it is designed to emphasize on mindfulness concepts. For instance, acceptance is a common concept between mindfulness and empathy [19]; therefore, the emphasis on such acceptance that can be obtained via mindfulness, empathy can be improved as well, and by fostering these two skills simultaneously, moral sensitivity can be improved among nurses, consequently.

### Limitation

Such concepts like moral sensitivity and empathy can be affected by cultural variables; in generalization of the study findings to other non-Iranian populations caution should be considered. On the other hand, due to the limitations of time and finance, some moderator variables were not controlled such as the ward that a nurse works there, work experience, and sex influencing on empathy or moral sensitivity; therefore, in findings generalization, these issues should be mentioned. In sum, for more emphasis on the casual relationship between the study variables, and the precise and practical appraisal of the study suggestions, it is highly recommended the effect of mindfulness and empathy skills teaching on nurses’ moral sensitivity in further experimental researches should be testified. To better fit the model, 3 items of mindfulness scale were eliminated and discriminant validity of compassionate care subscale from empathy scale and moral strength subscale from moral sensitivity scale were not complete; which could be one of the weaknesses of the model. This could be due to using of short forms of scale rather than long forms. Measuring these variables with a short form of scale may not have been able to properly assess the characteristics of nurses. However, the researchers speculated that fewer questions might be better for participants to respond. However, in future research, it is better to use a larger number of samples and a long form of scales.

## Data Availability

Data and materials are confidential but they will be available upon reasonable request from the corresponding author.

## Abbreviations

SEM: Structural equation modeling
PLS: Partial least squares
CR: Composite reliability
AVE: Average variance extracted
GoF: Good of fitness
